# VITAL INVOLVEMENT: LINKING CREATIVITY TO LATER-LIFE WELL-BEING

**DOI:** 10.1093/geroni/igz038.1556

**Published:** 2019-11-08

**Authors:** Helen Q Kivnick

**Affiliations:** University of Minnesota School of Social Work, Saint Paul, Minnesota, United States

## Abstract

Erikson’s principle of Vital Involvement (VI) holds that psychosocial health in older adulthood rests on elders’ meaningful, reciprocal engagement with the world outside the self. Older adulthood’s focal tension between Integrity and Despair is fundamentally grounded in elders’ “…vital involvement, with life’s people, materials, activities, ideas, institutions, and so forth. [This engagement is] … every bit as important as …[the] reminiscence” (Kivnick &Wells, 2014) we have long identified as a path to wisdom. Through several empirical projects, our team has identified five dimensions of VI in older adulthood. Each is clearly invoked as an elder engages such arts media as clay, paint, paper, fiber, wood, words, music, movement, and more. These VI dimensions are also invoked in an elder’s creative engagement with the people, materials, activities, and institutions of everyday life. This presentation illustrates ways that VI, through these dimensions, both facilitates and also expresses psychosocial well-being in older adults.

